# A prototype of knowledge-based patient safety event reporting and learning system

**DOI:** 10.1186/s12911-018-0688-5

**Published:** 2018-12-07

**Authors:** Hong Kang, Sicheng Zhou, Bin Yao, Yang Gong

**Affiliations:** School of Biomedical Informatics, The University of Texas Health Science Center at Houston, 7000 Fannin St, Houston, TX 77030 USA

**Keywords:** Patient safety, Knowledge base, Information storage and retrieval

## Abstract

**Background:**

Patient falls, the most common safety events resulting in adverse patient outcomes, impose significant costs and have become a great burden to the healthcare community. Current patient fall reporting systems remain in the early stage that is far away from reaching the ultimate goal toward a safer healthcare. According to the Kirkpatrick model, the key challenge in reaction, learning, behavior and results is the realization of learning stage due to the lack of knowledge management, sharing and growing mechanism.

**Methods:**

Based on the key contributing factors defined by AHRQ Common Formats 2.0, a hierarchical list of contributing factors for patient falls was established by expert review and discussion. Using the list as an infrastructure, we designed and developed a novel reporting system, where a strategy to identify contributing factors is intended to provide reporters knowledge support, in the form of similar cases and potential solutions. A survey containing two scenarios was conducted to evaluate the learning effect of our system.

**Results:**

In both scenarios, potential solutions recommended by the system were annotated with correct contributing factors, and presented only when the corresponding factors were identified from the query report or selected by the user. The five experts show substantial consistency (Fleiss’ kappa > 0.6) and high agreement (ranging between fully agree and mostly agree) in the assessment of the three perspectives of the system, which verifies the effectiveness of the proposed knowledge support toward sharing and learning through the novel reporting system.

**Conclusions:**

This study proposed a profile of contributing factors that could measure the similarity of patient safety events. Based on the profile, a knowledge-based reporting and learning system was developed to bridge the gap between surveillance, reporting, and retrospective analysis in the fall management circle. The system holds promise in improving event reporting toward better and safer healthcare.

## Background

Patient safety events (PSE) are the most concerns in the improvement of healthcare quality [1]. With more than 251,000 (9.5%) annual deaths, PSE is ranked the third leading cause of death in the U.S. following heart disease and cancer [2, 3]. Among the PSE, patient falls are the most common events resulting in adverse patient outcomes and imposing significant costs, a great burden to the healthcare community. Patient Safety Organization (PSO) has listed patient falls as one of the top PSE [4]. The fall rate in acute-care hospitals is between 1.3 and 8.9, mainly ranging from 3 to 5 per 1000 occupied bed days [5]. A fall with injury adds in average 6.3 days to the hospital stay and costs around $14,000, which are a huge waste of time and money for both patients and healthcare facilities [6, 7]. Different from diseases, which could be effectively controlled in accordance with clinical procedures, patient falls and other PSE subtypes are difficult to control due to multiple inputs including healthcare providers, systems, or even patients [8].

About 92% in-hospital falls are preventable [9]. Prevention and assessment toolkits as well as reporting systems would enable safety specialists to analyze events, identify underlying factors, and generate actionable knowledge to mitigate risks [10–12]. The toolkits provide protocols for patient fall prevention in terms of leadership, evaluation of fall risks (vital status, medication, environments, etc.), patient education, and event rate reduction. Commonly used fall prevention and assessment toolkits include AHRQ WebM&M [13], AHRQ Patient Falls Prevention Toolkit [14], Joint Commission Center for Transforming Healthcare Targeted Solutions Tool [15], Pennsylvania Patient Safety Authority Tools [16], etc. PSE reporting systems have been implemented for collecting PSE data and conducting root cause analyses (RCA). Mandatory and voluntary reporting systems complement each other and serve different levels and purposes in the PSE management [17]. The Institute of Medicine (IOM) recommended patient safety reporting systems (PSRS) [18] for understanding why patients are harmed by healthcare [19]. AHRQ created the Common Formats (CF) [20] to standardize reporting formats and help healthcare providers uniformly report PSE. The CF fall-reporting form includes 13 structured questions covering key contributing factors of fall event reporting and RCA, such as circumstances, outcome, fall risk assessment, preventions, medication, and assistant devices [20]. Since 2000, at least 30 PSE reporting systems have been established in the U.S., the initiatives to improve patient safety based on the common belief that data supports learning from the events and creating actionable knowledge [21].

However, healthcare providers fail to receive timely feedback and customized knowledge support from current toolkits and systems [22, 23]. The current toolkits are isolated education manuals with no connections to reporting systems, which leads to a lack of effective and efficient interactions for shared learning among healthcare providers. In addition, most solutions provided by the toolkits are comprehensive suggestions that are easy to follow but less tailored to fit into the reporting scenarios [24]. Current reporting systems do not present any mechanism to guarantee reporting quality because of its voluntary nature and clinicians' waning enthusiasm due to no feedback. The systems will become redundant databases when low-quality reports dominate. As a result, the reports would not serve the sharing and learning purposes of event reporting recommended by IOM [25]. Despite PSO makes efforts to standardize reporting formats by using AHRQ CF, many reporting hospitals cannot upgrade or replace their own reporting mechanisms that have been in use for years, which poses a barrier for event data standardization. There has been no support for reporters to glean helpful information from previous cases and learn from the tips suggested by the toolkits due to the lack of connection among different PSE resources [26].

To bridge the gap between surveillance, reporting, and retrospective analysis stages in the patient safety event management circle [22], developing a learning-oriented reporting system is an urgent need. The system is expected to automatically identify event contributing factors, continually enrich and update the knowledge in the PSE domain, and provide timely knowledge support such as similar cases and recommended solutions before, during or after event reporting. The Kirkpatrick model [27] provides the technique for appraisal of the evidence for any reported training program and can be used to evaluate whether a training program meets the expected outcomes of both organizations and the staff [28]. The Kirkpatrick model has been discussed and proven as an effective tool guiding and evaluating the development of learning-oriented PSE reporting system [29]. The four-level Kirkpatrick model is organized from basic to advanced levels, i.e., reaction, learning, behavior, and results, each of which addresses a sub-goal that is necessary to achieve. Current PSE reporting systems mainly focus on the reaction level that is far away from improving reporters’ behavior toward safer healthcare. The key challenge resides in the realization of learning level due to the lack of knowledge management, sharing and growing mechanism. A well-organized contributing factor list could be an effective tool to build this mechanism. Unfortunately, the prevailing contributing factors for patient safety are either too general (e.g., AHRQ CF [20]) or too specific (e.g., Castro’s list of contributing factors on Health IT exclusively [30]), because clear annotation criteria were unavailable for users to follow. As a result, no further knowledge supports could be provided based on these contributing factors. To identify the contributing factors toward a knowledge base of patient safety events, a rule-based model could be applied since it shares the common cognitive process with a human.

In this study, we 1) proposed a hierarchical list of contributing factors for patient fall events, 2) developed a rule-based contributing factor annotation model for both structured and unstructured reporting data, and 3) developed a event reporting and learning system with customized knowledge support and feedback mechanism for patient falls. Our system promotes the event reporting system to the learning level of Kirkpatrick model that reporters can learn how to address causes of errors, and improve their engagement and patient safety knowledge. The system bridging the gap between the surveillance, reporting, and retrospective analysis levels in the management circle for patient falls is expected to improve event reporting through a novel management and learning framework for PSE toward better and safer healthcare.

## Methods

### PSO fall reports

The AHRQ CF [20] is a set of standardized questionnaire-based forms with nine subtypes defined by PSO, including blood or blood product, device or medical/surgical supply including health information technology, fall, healthcare-associated infection, medication or other substance, perinatal, pressure ulcer, surgery or anesthesia, and venous thromboembolism. Statistics from PSO show that fall events hold the most number of reports than those of other event types. In this study, we employed 1836 de-identified fall reports collected by our collaborative PSO during year 2016. Each de-identified report includes the structured fields (4 general questions shared by all event types and 13 specific questions for fall event exclusively) and the unstructured fields (event description and responses, such as other, in free texts in the structured fields).

### Establish contributing factors and a rule-based annotation strategy

The AHRQ CF provides a document “Generic-National Collection (core)” [31], which summarized the core data elements required for PSE reporting at the local level by healthcare providers to PSOs and the PSO Privacy Protection Center (PSOPPC) for national aggregation and analysis. The elements include the *type of patient safety concern*, the *circumstances of event* of unsafe condition, *patient information*, and *reporter information*. As a sub-section of *circumstances of events*, the section of *contributing factors* has nine factor categories including 42 factor terms, which were applied as the infrastructure of fall contributing factors. Three domain experts who are familiar with patient safety data and have event reporting experiences reviewed 1469 PSO fall reports, accounting for 80% of the total in year 2016. The expert review responsibilities included:

1. Extend the hierarchical list of contributing factors with new identified factors.
2. Annotate each report with contributing factors, i.e., factors identified from the structured fields and the unstructured fields were annotated separately.
3. Highlight keywords in the unstructured fields contributing to the factor identification.
4. Label each option of the structured fields with one or more contributing factors.

The experts reviewed individually. Group discussions were held to resolve divergences and reach final decisions. New factors were added as follows:

1. Terms of diseases and symptoms were coded by the International Classification of Diseases, Tenth Revision, Clinical Modification (ICD-10-CM) [32].
2. Terms of surgeries were coded by Current Procedural Terminology (CPT) [33].
3. Terms of medications were coded by the Anatomical Therapeutic Chemical Classification System with Defined Daily Doses (ATC/DDD) [34].

### Create identification rules for contributing factors

A super inspector, in addition to the three domain experts, reviewed the annotation results and assigned regular expressions to each paired contributing factor and corresponding keywords. A regular expression is a sequence of characters typically used in rule-based models to define a search pattern. A regular expression was further labeled as either “true” or “false” as activation status of contributing factor when the expression is matched. For example, one expression of contributing factor “bed alarms” was coded as “\b (forgot)\b.+\b (alarm)\b.+\b (back on)\b” and labeled as “true” because “bed alarms” could be a contributing factor if a nurse forgot to reset the alarm. Another expression of factor “fatigue” was coded as “\b (denied fatigue)\b” and labeled as “false” because “fatigue” should not be a factor if a patient denied that. Each expression was called an identification rule for the corresponding contributing factor. Thus, each factor may have more than one identification rules. For the structured fields, the identification rules were coded by listing all activation options of the questions to each contributing factor.

The remaining 367 (20%) reports were applied for evaluation, within which five high-quality reports with little factor overlaps were reserved for the evaluation of the knowledge support strategy, and the rest 362 reports were applied to evaluate the identification rules of fall event contributing factors. The rules were run on each report to identify the contributing factor(s), after which the experts reviewed and scored the factor(s) independently with four Likert scales [35], i.e., 1) *fully agree*, 2) *mostly agree*: lacking necessary factor(s), 3) *mostly disagree*: appearing some incorrect or inaccurate factor(s), and 4) *fully disagree*. Group discussions were held to generate final decisions when divergences appeared.

### Develop identification rules for event solutions

In our previous research [24], we collected and categorized 122 fall solutions from multiple resources. The solutions covered almost all aspects in fall prevention, such as assistive devices, environment and equipment, fall event reporting, use of fall risk assessment tools, individual patient fall risks, medications, patient and family education, and rounding. To incorporate the solutions in the knowledge support mechanism, the three domain experts further reviewed and labeled each solution to be associated with at least one contributing factor. Group discussions were held to generate final decisions when divergences appeared.

### Develop and evaluate a rule-based knowledge support strategy based on contributing factors

**Fig. 1:**
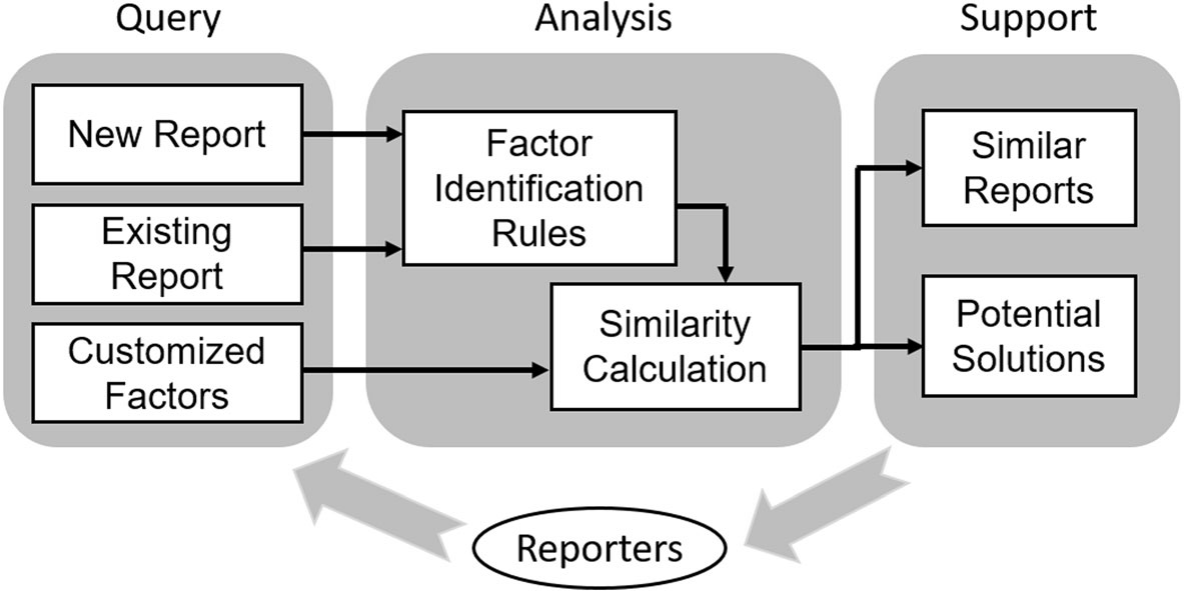
A rule-based knowledge support strategy for event reporting

A knowledge support strategy was developed based on the identification rules of fall event contributing factors. As shown in Fig. 1, the strategy includes three modules, i.e., query, analysis, and support. A learning session can be initialized by determining a query, either an event report (new report or existing report) or a group of customized contributing factors. A report query is screened by all identification rules and annotated with identified contributing factors, while the customized factors skip the screening process. Then the contributing factors of query are compared with the factors of all the other reports in the database.

As shown in Eq. 1, a similarity score *S*_*qi*_ is calculated between the query *q* and each of the other reports *i* by compacting the annotations into binary vectors (*Q* and *V*_*i*_) and measuring the cosine of two vectors (vector space [36]).

1 $$ {S}_{qi}=\frac{Q\bullet {V}_i}{\left\Vert Q\right\Vert \left\Vert {V}_i\right\Vert } $$

If the query is a group of customized factors, the similarity score *S*_*qi*_*’* is *S*_*qi*_ plus the number of same factors *N*_*qi*_ between the query and report *i* (Eq. 2).

2 $$ {S}_{qi}^{\prime }={S}_{qi}+{N}_{qi} $$

This similarity calculation method has been proven effective on PSE reports in our previous study [37]. As a result, the knowledge support module provides 10 most similar reports based on the similarity scores, and potential solutions determined by the identical contributing factors with the query. These materials are supposed to be a customized knowledge support to reporters toward a case-based learning mechanism.

We designed two scenarios to evaluate the learning effect of our strategy. The first one is applying the 5 reserved reports as query samples, which are not involved in the design and evaluation of the identification rules. The other scenario is applying a group of user customized contributing factors as the query. Five PSO experts participated in the evaluation. After reviewing the scenario-based learning materials (i.e., similar reports and recommended solutions), the participants were asked to complete a survey (Table 1).

**Table 1:** Survey for learning effect evaluation

*Scenario 1* *(report queries)* Q1a. Do you think the query and its top three similar reports were annotated with correct contributing factors? Q2a. Do you think the scenarios of the three reports were similar to that of the query report?	
*Scenario 2* *(contributing factor queries)* Q1b. Do you think the top three recommended reports were annotated with correct contributing factors? Q2b. Do you think the scenarios of the three reports matched your expectation?	
*Both Scenarios* Q3. Do you think the three similar/recommended reports and the solutions provided strong knowledge support for learning purpose? Q4. Is there any other comment you would like to make?	

Q1-Q3 are single-choice questions with four scaled choices: 1) *fully agree*, 2) *mostly agree*, 3) *mostly disagree*, and 4) *fully disagree*, while Q4 is a subjective question. Participants reviewed the materials and completed the survey individually. A Fleiss’ kappa, a statistical measure for assessing the reliability of agreement among multiple raters, was calculated to the answers of Q1-Q3 from the five participants. To simplify calculation, *fully agree* and *mostly agree* were treated as *agree*, while *mostly disagree* and *fully disagree* were treated as *disagree*

### Apply user-centered design principles to a knowledge-based reporting and learning system

User-centered design (UCD) has been proven effective in improving reporting accuracy, completeness, and timeliness [38]. We applied UCD features such as input validator, user-friendly layout, role-based operation, and user feedback, to the development of the knowledge-based reporting and learning system. The evaluation of UCD features were not included in this study.

## Results

### A hierarchical list of contributing factors for fall events

Based on the infrastructure (including 9 factor categories and 42 factor terms) derived from AHRQ CF, we extended the contributing factor list for fall events to 14 categories and 195 terms by reviewing the 80% fall event reports. The maximum depth of the hierarchical structure is five. The categories are shown in Table 2.

**Table 2:** A summary of the hierarchical contributing factor list for fall event reports

Index	Categories	Num. of Terms	Max Depth
1	Communication, other than at the time of handover/handoff	4	2
2	Handover/handoff	1	1
3	Data issues (e.g., availability, accuracy)	4	2
4	Environment (e.g., culture of safety, physical surroundings)	18	3
5	Human factors (e.g., fatigue, stress, inattention, cognitive factors)	87	5
6	Policies and procedures, including clinical protocols (e.g., absence, adequacy, clarity)	6	3
7	Staff qualifications (e.g., competence, training)	3	2
8	Supervision/support (e.g., clinical, managerial)	3	2
9	Health Information Technology (HIT)	8	2
10	Medications	39	5
11	Consequences	10	3
12	Admission/discharge	3	2
13	Event location	5	2
14	Therapy prior fall	4	2

### A rule-based strategy for annotating contributing factors

For the unstructured fields, a regular expression was labeled as either “true” or “false” depending on the activated or deactivated status of certain contributing factor when matching the expression. As a result, 939 rules were coded, including 862 activation rules and 77 deactivation rules. For the structured fields, 43 out of 195 contributing factors were coded with at least one answer option in the AHRQ CF fall reports. 362 reports out of the remaining 20% fall event reports were applied to evaluate the factor identification rules. As shown in Table 3, 349 (96.4%) reports were scored as *fully agree* by the expert group, which indicates the 939 rules can effectively identify fall contributing factors from both structured and unstructured reports.

**Table 3:** Distribution of the scaled scores -- Results of evaluating the identification rules

Scaled Scores	1: Fully Agree	2: Mostly Agree (lacking factor(s))	3: Mostly Disagree (appearing wrong factor(s))	4: Fully Disagree
Reports (*N* = 362)	349 (96.4%)	7 (1.9%)	6 (1.7%)	0

The fall solutions proposed in our previous study were further annotated with fall contributing factors by domain experts. 150 of 195 contributing factors were covered by a total of 122 solutions after coding experts’ annotation results (one solution may cover more than one factor).

### A knowledge-based reporting and learning system

**Fig. 2:**
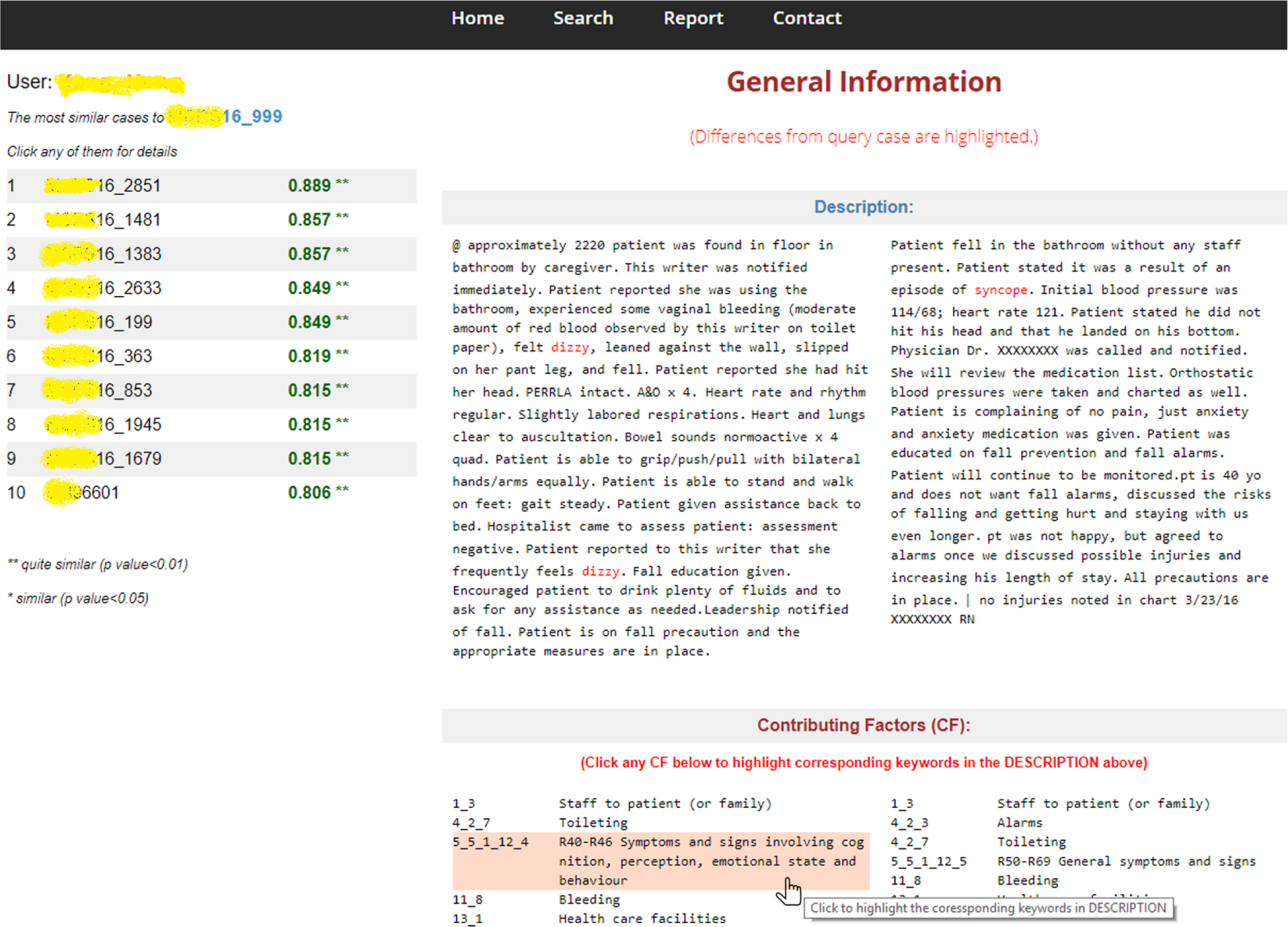
A screenshot of similar report sorted by the similarity scores in a descending order. When a report is selected as a query (*scenario 1*), top 10 similar reports will be displayed on the left side of the page. After clicking any of the 10 similar reports, corresponding details will be shown on the right side. The selected similar report is presented side by side with the query report. All contributing factors are identified and listed under the description sections. By clicking any factor entry, the keywords contributed to the identification will be highlighted in red within the description

**Fig. 3:**
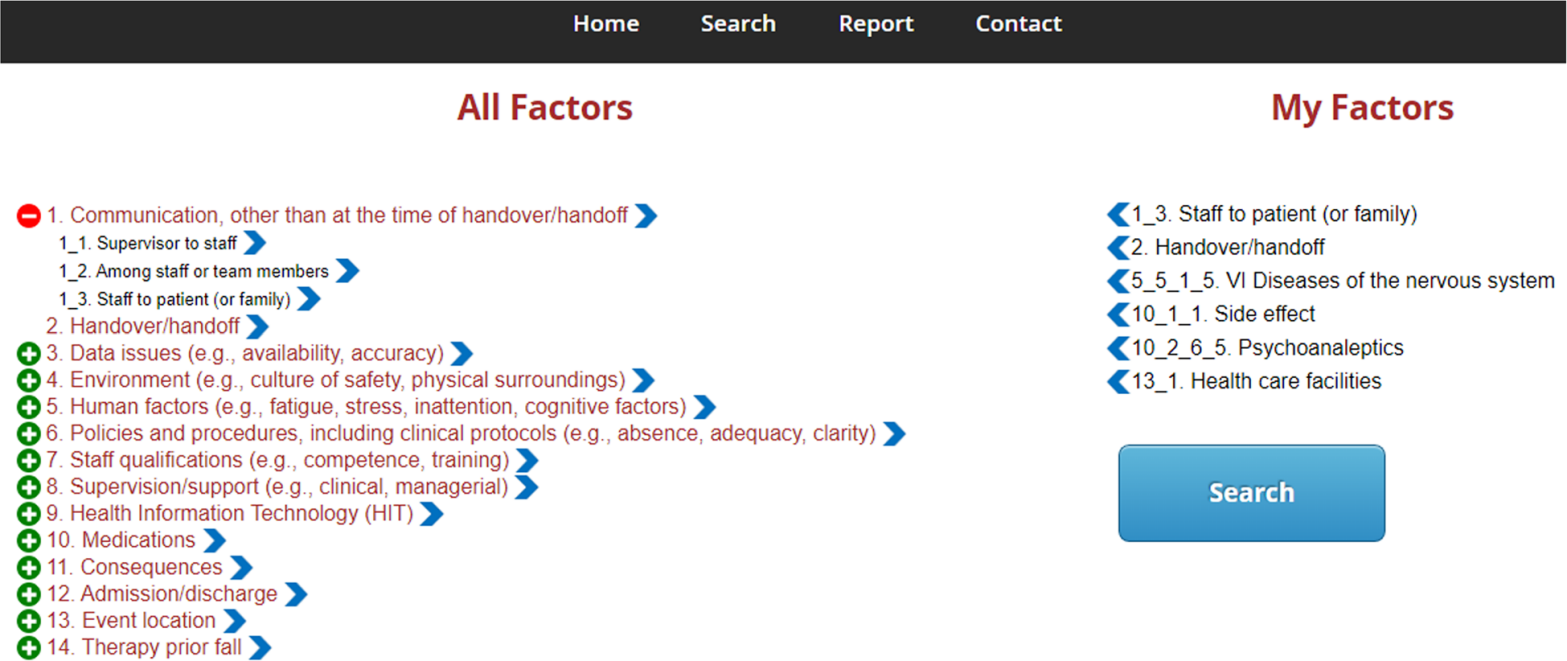
A screenshot of customized contributing factor. Rather than applying an event report as a query, the user can also directly select the contributing factors and to launch the similarity search. The user is free to include/exclude any of the total 195 factors to/from “My Factors” and launch a similarity search. The calculation of similarity scores is based on Eq. 2, and result display page is referred to Fig. 2

A knowledge-based reporting and learning system was developed by applying the contributing factor identification rules and the corresponding similarity measurement strategy. The current version runs on a local webserver developed by JSP and MySQL. UCD features such as input validator, user-friendly layout, role-based operation, and user feedback were incorporated into the system. To assess the knowledge support mechanism, we simulated two learning scenarios. In *scenario 1-learning after reporting or browsing*, by applying the report submitted or selected by the user as a query (Fig. 2), while in *scenario 2-active learning*, the query was a group of contributing factors selected by the user (Fig. 3).

**Fig. 4:**
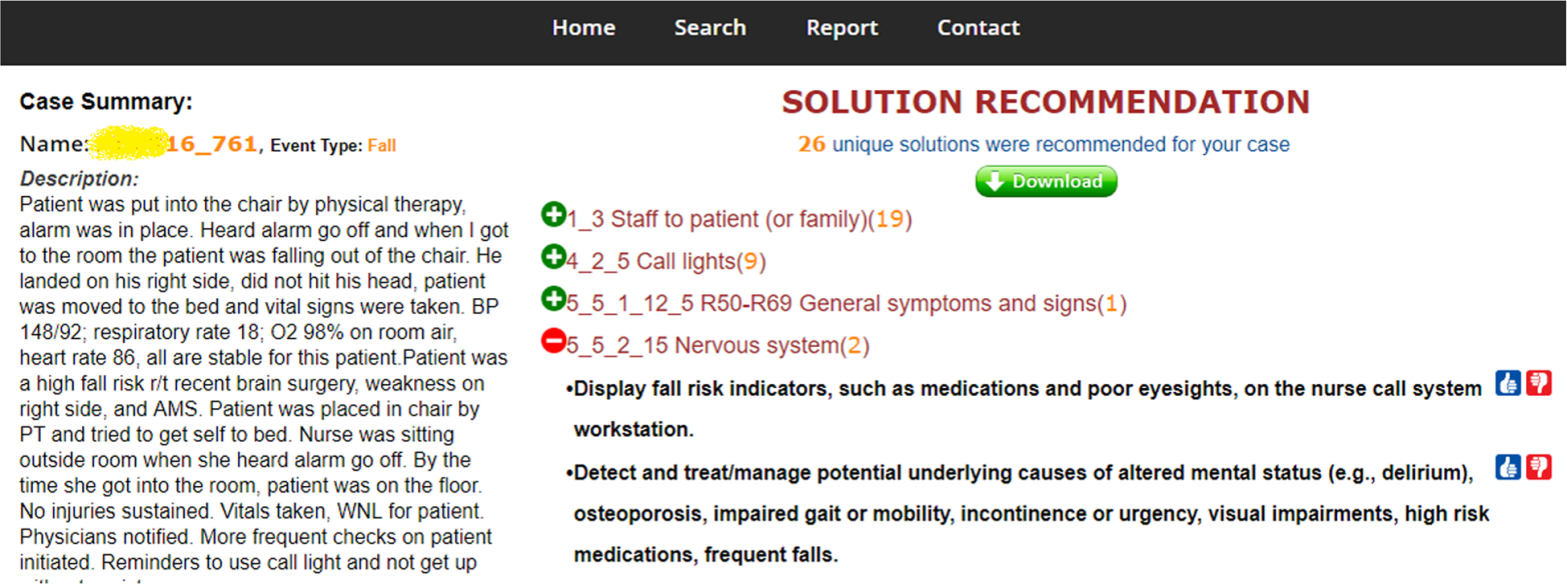
A screenshot of the solution recommendation

Recommending solutions is an essential knowledge support for both scenarios. As shown in Fig. 4, each solution was annotated with contributing factors, and was presented only when the corresponding factor(s) was/were identified from the query report (*scenario 1*) or selected by the user (*scenario 2*). The ranking order of the factors were initialized randomly and would be optimized according to user preferences collected from the thumb up/down buttons. By clicking the download button, the user could get more information about the identified solutions, including solution category, source, link, and all related contributing factors.

To evaluate the knowledge support mechanism in both scenarios, a survey was conducted among five patient safety experts working with a PSO. Q1~Q3 were designed to assess contributing factor identification, similarity search, and learning effect respectively. The scaled scores in Table 4 were calculated by averaging the scores from the five experts, while Fleiss’ Kappa [39, 40] was calculated for the scores of each question to measure the consistency among experts. The five experts show substantial consistency (Fleiss’ kappa > 0.6) in the assessment of the three perspectives of the system. Almost all mean scores are lower than 2, indicating a consensus of agreement of the effectiveness of the proposed functions toward learning. The only exception is the similarity assessment in scenario 2, where the mean score is 2.2. The reason was indicated in the experts’ comments that sometimes no report in current database covers all contributing factors selected by expert, which could lead to a bias in the similarity measurement. This bias is thought to be mitigated as the database grows.

**Table 4:** The result of survey-based evaluation for the knowledge support strategy

	Mean Score (*N* = 5)						Fleiss’ kappa	*P*-value
	Scenario 1					Scenario 2		
	Report1	Report 2	Report3	Report4	Report5			
Q1 (assess factors)	1.4	1.4	1.6	1.6	1.6	1.2	0.68	< 0.01
Q2 (assess similarity)	2.0	1.8	1.6	1.8	2.0	2.2	0.61	< 0.01
Q3 (assess learning)	1.4	1.4	1.8	1.4	1.8	1.6	0.66	< 0.01

*Please refer to method section for the content of each question

*Scaled scores: 1. fully agree; 2. mostly agree; 3. mostly disagree; and 4. fully disagree

*Fleiss’ kappa between 0.61 and 0.80 indicates a substantial consistency among multiple subjects

## Discussion

### Bridging the gap between reporting and learning

The increasing number of PSE and needed solutions pose a challenge for exploring the potential connections among the events and presenting the events in an organized view in a timely manner. Current reporting systems do not have robust processes for analyzing and acting upon aggregated event reports. The expected knowledge support through event reporting is not manifest owing to many self-perceived barriers to voluntary reporting, such as no feedback, lengthy reporting forms competing with other priorities, and observed events that may seem “trivial”. A well-organized contributing factor list could be a reasonable start point that serves as an effective tool to manage knowledge. Nonetheless, none of the contributing factors relating to patient safety events has clear annotation criteria for reporters and researchers to follow. Focusing on fall events, we proposed a hierarchical list of contributing factors and annotation rules by reviewing PSO reports, and based on which we developed a mechanism that integrates event reports and solutions toward case-based knowledge support. This learning-oriented mechanism bridges the gap between the reaction (reporting) and learning defined by the levels in the Kirkpatrick model, and paves the way for realizing the behavior and results at the higher levels.

### Integrating and balancing unstructured and structured reports

Structured data refers to highly organized information, so that its inclusion in a relational database is seamless and readily searchable by simple, straightforward search engine algorithms or other search operations; unstructured data is the opposite. Further, structured data is akin to computer language, which makes information easier to use with computers; while unstructured data is easier for human users, who do not interact with information in a strict database format [41]. However, experts estimate that 80% of the world’s data is unstructured, which means researchers that do not access this data are losing much useful information [42]. In clinical settings, free-text reporting (unstructured) is preferred by healthcare providers over form-based reporting (structured) for PSE, because the providers are less acquainted with the categories and fields pre-defined by the form-based reporting. The lack of connection between structured and unstructured data could be overcome by our rule-based strategy for identifying contributing factors since no pre-knowledge requirement about PSE taxonomy is needed for using our system. The timely knowledge support provided to targeted users can go beyond reporting format and holds promise in improving system interoperability and data sharing between sites, firms and industry sectors.

### Minimizing the implementation cost

The hierarchical list of contributing factors for fall was established based on AHRQ CF and expert review, and could also support other PSE types through case-based modification and extension. The knowledge-based reporting and learning system in this study can be directly applied by PSO since it was developed based on AHRQ CF. PSO can provide an interface to local hospitals for reporting or learning. Therefore, the learning can occur at three levels. At the individual level, healthcare providers will have time to report and learn from what is being reported or has been reported. At the group level, for example, in a certain clinical department, the collective knowledge gleaned from the entire team could be shared through the system. The contributing factors in the events could be analyzed by an automatic process for patient safety experts to review and confirm as feedback to the healthcare providers. At the organizational level, PSO can compare and analyze the reports across all reporting hospitals and provide tailored recommendations or solutions. Our system will not consume more time in reporting but add on learning features at the individual level. Our system is expected to save time and effort at the group and organizational level in creating feedback and analyzing contributing factors. For the hospitals having their own reporting systems, the contributing factor identification rules could be extracted as a natural language processing plug-in and embedded into the existing systems to minimize the implementation cost.

### Detecting fall risks in EHR and CPOE systems

During the expert review, we found that the contributing factors to patient falls proposed in this study are sufficient to covere most fall scenarios in the PSO reports. We also found that diseases, surgery histories, and medications are essential contributing factors to patient falls. For example, the patients who were diagnosed as seizures, had neurosurgery, or took muscle relaxants, have higher fall risks than regular patients. Therefore, EHR and computerized physician order entry (CPOE) systems have great potential in detecting the fall cases and the corresponding contributing factors since a large amount of fall risks regarding to diseases, surgery histories, and medications are ignored. On the other hand, applying the factor identification tool in EHR and CPOE systems will facilitate the detection of fall risks at early stages, which could reduce unnecessary bed occupation days and save patient and healthcare facility’ cost due to in-patient fall events.

### Limitations

All evaluations in this project were processed through expert review since there is no gold standard for PSE similarity measurement and solution recommendation. Each expert might bring a different perspective that may result in biases toward the learning effect assessment limited by the small sample size of survey (*N* = 5). The variety of the one-year data may be limited and insufficient to substantiate the rules of contributing factors, which could impact the performance of similarity measurement.

### Future work

The factor identification rules will be extended by increasing the sample sizes of  fall reports and PSO experts in the evaluation survey. The effectiveness and efficiency of UCD features will be initially evaluated through usability inspection and user evaluation.

## Conclusion

We developed a hierarchical list of contributing factors for patient falls and a rule-based factor identification model for both structured and unstructured reporting data. Based on the factors, a knowledge-based event reporting and learning system was developed to provide targeted knowledge support and feedback. The knowledge support includes the similar reports sharing contributing factors to the query, and recommended solutions to prevent the recurrence and serious consequences. The evaluation result indicates that a profile of contributing factors could measure the similarity of patient safety events and organize patient safety knowledge for shared learning. As a learning-oriented platform, our system is expected to help healthcare professionals gain better understanding of PSE and actionable knowledge within their clinical workflows.

## Abbreviations

AHRQ: Agency for Healthcare Research and Quality
CF: Common Formats
PSE: patient safety event
PSO: Patient safety organization
RCA: root cause analyses
UCD: user-centered design
